# Supplementary material for: Identification and Evaluation of qRT-PCR Reference Genes in Melanaphis sacchari
Source: Insects. 2024 Jul 11;15(7):522. doi: 10.3390/insects15070522 (PMC11277337; doi:10.3390/insects15070522)

Supplementary file S1

The following sequences are sequencing comparisons of candidate internal reference genes

1. Actin >XM_025350371.1 (original sequence)

ATGGATGACGATGTTGCGCCTTTGGTTGTCGACAACGGCTCTGGTATGTGCAAGGCTG
GTTTCGCCGGCGATGACGCACCCCGTGCTGTCTTCCCGTCCATTGTAGGCAGACCACG
TCATCAAGGTGTTATGGTCGGTATGGGACAAAAGGATAGCTACGTAGGAGATGAAGCC
CAAAGCAAACGTGGTATCCTTACTTTGAAATATCCCATTGAACACGGAATCATCACCAA
CTGGGATGATATGGAGAAAATATGGCATCATACTTTCTACAATGAACTTCGTGTTGCCCC
AGAAGAACATCCAACACTGTTGACCGAAGCCCCATTGAATCCAAAGGCTAACCGTGA
AAAGATGACCCAAATTATGTTTGAAACTTTCAACACTCCTGCTATGTATGTTGCCATCCA
AGCTGTATTATCTTTATACGCTTCTGGCCGTACCACTGGTATTGTTTTGGACTCAGGTGA
CGGTGTCTCCCACACTGTTCCCATCTATGAAGGTTATGCATTACCTCACGCTATTCTTCG
TTTGGACTTAGCTGGTCGTGACTTGACCGACTACTTAATGAAAATCTTGACCGAGAGA
GGTTACAGCTTCACCACTACTGCTGAGCGTGAAATCGTCCGTGACATCAAAGAAAAAT
TATGTTATGTCGCTCTGGACTTCGAACAGGAAATGGCCACAGCCGCCGCTTCTACTTCC
TTAGAGAAATCCTATGAATTACCTGACGGACAAGTCATAACCGTCGGTAACGAACGTTT
CCGTTGCCCAGAATCCTTGTTTCAACCATCATTTTTGGGAATGGAATCTTGCGGTATCCA
CGAAACTGTATATAACTCCATCATGAAATGTGACGTTGACATCAGAAAGGACTTATACG
CTAACACTGTACTTTCTGGAGGTACAACTATGTACCCAGGAATTGCTGACAGAATGCAA
AAGGAAATTACCGCATTGGCTCCTAGCACAATCAAGATTAAGATTATTGCCCCACCAGA
ACGTAAATACTCCGTATGGATCGGTGGTTCCATCTTAGCTTCTCTATCTACCTTCCAACA
AATGTGGATCTCTAAACAAGAATACGATGAATCCGGTCCAGGAATTGTTCACCGTAAAT
GTTTTTAA

Actin (Sequencing results)

CTGGTCGGTATGGGACAAAAGGATAGCTACGTAGGAGATGAAGCCCAAAGCAAACGT
GGTATCCTTACTTTGAAATATCCCATTGAACACGGAATCATCACCAACTGGGATGATATG
GAGAAAATATGGCATCATACTTTCTACAATGAACTTCGTGTTGCCCCAGAAGAACATCC
AACACTGTTGACCGAAGCCCCATTGAATCCAAAGGCTAACCGTGAAAAGATGACCCA
AATTATGTTTGAAACTTTCAACACTCCTGCTATGTATGTTGCCATCCAAGCTGTATTATCT
TTATACGCTTCTGGCCGTACCACTGGTATTGTTTTGGACTCAGGTGACGGTGTCTCCCA
CACTGTTCCCATCTATGAAGGTTATGCATTACCTCACGCTATTCTTCGTTTGGACTTAGC
TGGTCGTGACTTGACCGACTACTTAATGAAAATCTTGACCGAGAGAGGTTACAGCTTC
ACCACTACTGCTGAGCGTGAAATCGTCCGTGACATCAAAGAAAAATTATGTTATGTCGC
TCTGGACTTCAAACAGGAAATGGCCACAGCCGCCGCTTCTACTTCCTTAGAGAAATCC
TATGAATTACCTGACGGACAAGTCATAACCGTCGGTAACGAACGTTTCCGTTGCCCAG
AATCCTTGTTTCAACCATCATTTTTGGGAATGAAATCTTGCGATCCCCCCAAAAACTTTA
ACGATTTCTTCTTCAATGCGAAGTGTACACGTCAGAATGACACTTAGACTCTAACACTG
ACCTTTGCTGAATACGCAACTATAAACAGGTATCTAGCTAACGAATGTCCAAAAGATCT
CGCCATTGCGCCTCTGCCACAATCAATATCAATATCTATGCCCCTCCAACCGTATACCTC
ATATTGCATCGTAAATCGTA

Sequence comparison results

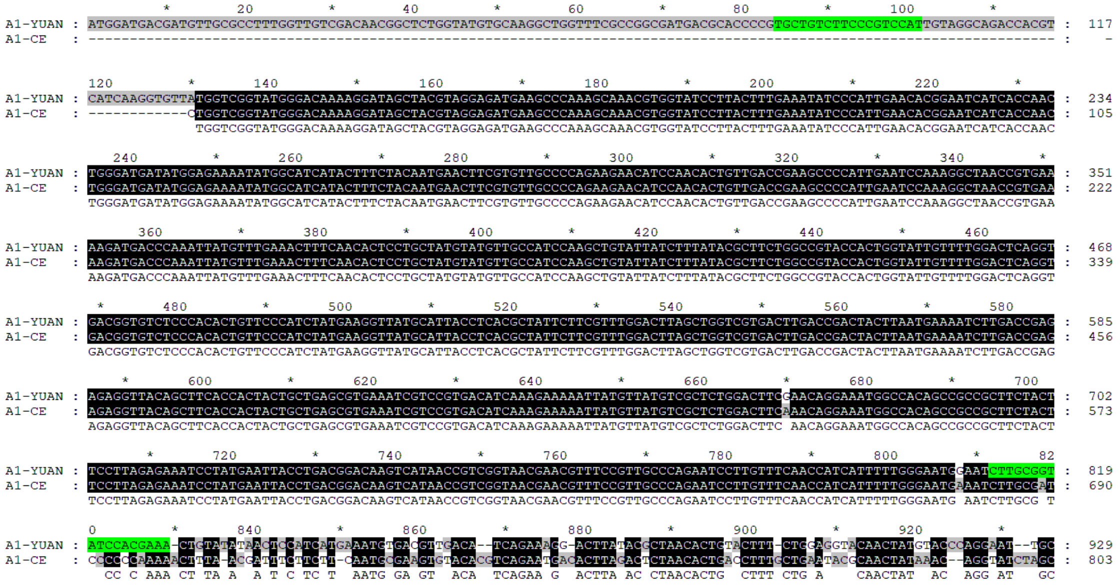

2. 18S ribosomal（18S） (original sequence)

>XM_025343011.1

ATGTCTCGTCCAGAACATTTAGCTCCACCAGAAATCTTTTATAATGATGATGAAGCTCG
AAAGTATACCCAAAATTCTCGAATGATTAATATTCAATTAGAAATGGCTGAAAGGTGTAT
GGAATTGTTGATGTTGCCCGAAGACGAACCATGTTTGCTATTAGATTTAGGTTGTGGTT
CTGGTCTAAGTGGCAGTGTTGCTGAAGATTCTGGTCATGTTTGGTTTGGAATGGACATT
TCAGACTCCATGTTGAGAGTAGCAAAAGAAAGAGAAGTCGAAGGTGATTTATTGTTAG
GAGACATTGGTGATGGCGTTCCATTTCAACCTGGTACATTTGATGGTGCATACAGTGTA
AGCACATTACAATGGTTATGTAATGCAGATAAATCATCACATAAACCTGCTAAAAGGTTA
TATACTTTGTTTTCATCATTATTAGCTTGCTTGGGACGCAACGCTAGAGCAGTCTTTCAA
TTTTATCCAGAAAATGTACATCAAACAGATTTGGTTGTATCACAAGCGAGAAAAGCTGG
ATTTTTTGGAGGTTTGTTGGTTGACTTTCCTGATAGTACTAAAGCAAAGAAATACTTTCT
TGTTTTGATGACTGGTGGTGCTATGCCTATGCCTGCAGCACTTGGTACAGAAAATTCAC
AAATTAATTATACATCAAAAAGAGAAAAAATGACAAAAGGGCGTGCATCAAAATTAGT
CAAAAAAAGTAGAGAATGGATTTTAGAAAAAAAAGAAAGGAGAAGACGTCAAGGAA
GAACTACTAGAGAAGATACCAAGTACACAGGACGATCAAGGAGTGGACGATTTTAA

18S ribosomal（18S）(Sequencing results)

GGTGGGTGGATGGAATTTCAGACTCATGTTGAGAGTAGCAAAAGAAAGAGAAGTCGA
AGGTGATTTATTGTTAGGAGACATTGGTGATGGCGTTCCATTTCAACCTGGTACATTTGA
TGGTGCATACAGTGTAAGCACATTACAATGGTTATGTAATGCAGATAAATCATCACATAA
ACCTGCTAAAAGGTTATATACTTTGTTTTCATCATTATTAGCTTGCTTGGGACGCAACGC
TAGAGCAGTCTTTCAATTTTATCCAGAAAATGTACATCAAACAGATTTGGTTGTATCAC
AAGCGAGAAAAGCTGGATTTTTTGGAGGTTTGTTGGTTGACTTTCCTGATAGTACTAAA
GCAAAGAAATACTTTCTTGTTTTGATGACTGGTGGTGCTATGCCTATGCCTGCAGCACT
TGGTACAGAAAATTCACAAATTAATTATACATCAAAAAGAGAAAAAATGACAAAAGGG
CGTGCATCAAAATTAGTCAAAAAAAGTAGAGAATGGATTTTAGAAAAAAAAGAAAGG
AGAAGACGTCAAGGAAGAACTACTAGAGAAGATACCAAGTACACAGGACGACAAAA

Sequence comparison results

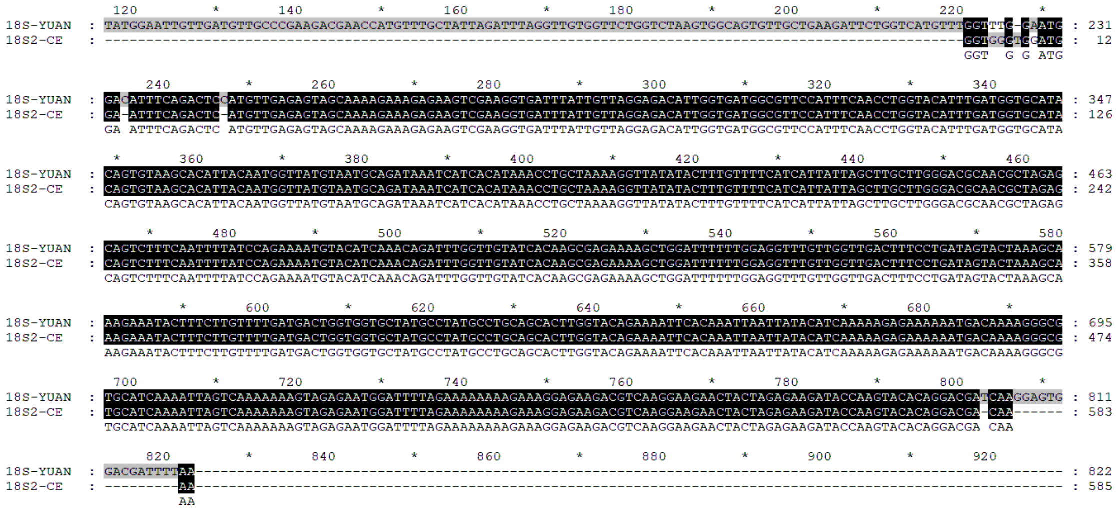

3. Elongation factor 1-alpha(EF-1α) (original sequence)

>XM_025346188.1 (elongation factor 1-alpha)

ATGGGTAAAGAAAAGGTACATATTAACATTGTTGTCATTGGACACGTCGATTCCGGTAA
ATCAACAACAACCGGTCACTTGATCTACAAATGTGGTGGTATCGACAAGCGTACAATTG
AAAAATTCGAGAAAGAAGCCCAAGAAATGGGTAAAGGTTCTTTCAAATACGCATGGGT
ATTGGACAAACTGAAGGCTGAACGTGAACGTGGTATCACTATTGATATTGCTTTATGGA
AATTCGAAACTGCCAAATACTACGTCACAATCATTGACGCACCTGGACACAGAGATTT
CATCAAGAACATGATCACTGGTACTTCCCAGGCTGATTGTGCTGTGCTTATTGTCGCTG
CTGGTACTGGTGAATTTGAAGCTGGTATTTCTAAAAATGGACAAACCCGTGAGCACGC
TCTATTGGCCTTCACTTTGGGTGTGAAACAATTGATTGTTGGTGTGAACAAGATGGACT
CCACTGAACCACCATACAGCGAAGCTCGTTTCGAAGAAATTAAGAAAGAAGTCAGCA
GTTACATCAAGAAAATTGGTTACAATCCAGCTGCTGTAGCTTTCGTTCCCATCTCTGGAT
GGAATGGAGACAACATGTTGGAAGTTTCCGACAAGATGTCATGGTTCAAAGGATGGAA
TATTGAACGCAAAGAAGGAAAGGCTGACGGTAAATGTCTTATTGAAGCTTTAGACGCT
ATCCTACCACCCAGTCGTCCAACTGACAAGGCTCTCCGTCTCCCACTCCAGGACGTCT
ACAAAATTGGTGGTATTGGAACAGTCCCAGTTGGTCGTGTTGAAACTGGTCTTTTGAA
ACCCGGTATGGTTGTGGTCTTTGCACCTGCTAACATCACCACTGAAGTTAAGTCTGTAG
AAATGCACCACGAAGCTTTGGTAGAAGCTGTCCCCGGAGACAACGTTGGTTTCAACGT
AAAGAACGTTTCAGTTAAAGAATTGAGACGTGGTTTCGTTGCTGGAGACACTAAGAAC
AATCCACCCAAGGGTGCTGCTGATTTCACTGCCCAGGTCATAGTATTGAACCACCCTGG
TCAAATTTCCAATGGATACACTCCAGTGTTGGATTGCCACACAGCCCATATTGCTTGCA
AATTCGCAGAAATCAAAGAGAAGTGTGACCGTCGTACTGGTAAAACTACTGAAGCTAA
TCCAAAGGCCATCAAATCTGGAGATGCTGCCATCATTACCTTGGTACCATCCAAGCCTT
TGTGTGTTGAAGCTTTCTCAGAGTTCCCTCCCTTGGGACGTTTTGCTGTACGTGACATG
AGGCAAACTGTTGCTGTTGGTGTCATCAAGAGTGTTAACTTCAAAGATCCGTCTGCCG
GCAAAGTAACAAAGGCCGCTGAAAAGGCCCAAAAGAAGAAATGA

Elongation factor 1-alpha(EF-1α) (Sequencing results)

AGGTTGTGGTCTTTGCACCTGCTAACATCACCACTGAAGTTAAGTCTGTAGAAATGCAC
CACGAAGCTTTGGTAGAAGCTGTCCCCGGAGACAACGTTGGTTTCAACGTAAAGAAC
GTTTCAGTTAAAGAATTGAGACGTGGTTTCGTTGCTGGAGACACTAAGAACAATCCAC

CCAAGGGTGCTGCTGATTTCACTGCCCAGGTCATAGTATTGAACCACCCTGGTCAAATT
TCCAATGGATACACTCCAGTGTTGGATTGCCACACAGCCCATATTGCTTGCAAATTCGC
AGAAATCAAAGAGAAGTGTGACCGTCGTACTGGTAAAACTACTGAAGCTAATCCAAA
GGCCATCAAATCTGGAGATGCTGCCATCATTACCTTGGTACCATCCAAGCCTTTGTGTG
TTGAAGCTTTCTCAGAGTTCCTTCC

Sequence comparison results

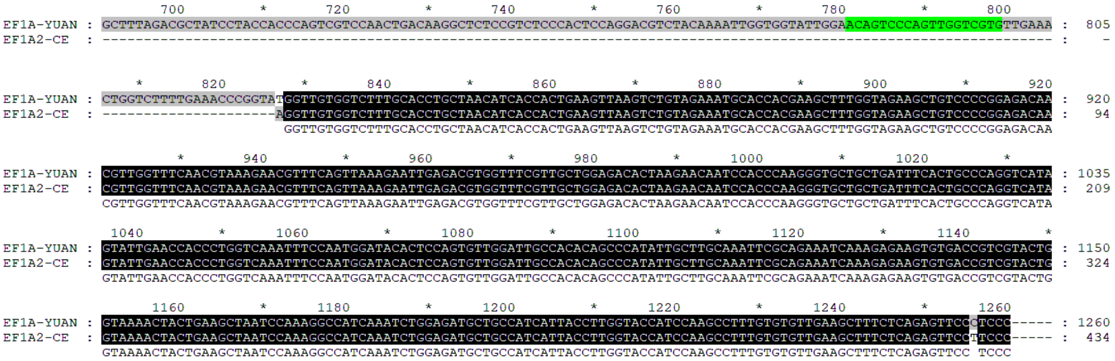

4. elongation factor 1-beta（EF-1β）(original sequence)

>XM_025337882.1

ATGGCTGCCGTTGACTTAAAAACTCCACAAGGCGTACAGTTCCTCGAAAACTACTTGA
GCCAGAAAAGTTACATTGTTGGATATGAACCATCCAAAGCCGACGTTGATACTTTTGCT
GCGATTCAAGCACCCCAGGCCAAGACTCCTAATGTGCTCAGGTGGTACAACCACATCA
AGTCGTTCACTGAGAAAGAGCGCACCCAGTTCCCAAACAAAAAAGCTGAGTTTTCAG
TGTCTTCGGCTAATGCTGCAAAACCGGCAGATGACGATGATGACGATGTAGATTTGTTC
GGTTCTGATGATGAAGATGATGAAGAAGCAGAACGTATCAAACAAGAAAGACTAAAG
GCTTATGCTGAAAAGAAAGCCACCAAAAAAGTAATTATCGCCAAGAGTAGCATTGTAC
TTGATGTTAAACCATGGGATGATGAAACCGATATGAAACAATTAGAAACACAAGTCCGA
TCCATTAACATGGATGGTTTAGTGTGGGGAGCTTCAAAATTAGTCGAGATTGCTTTTGG
TATCAAGAAGCTTCAAATTATGTGTATTGTAGAAGATGATAAAGTATCTGTCGATGCTCT
TACAGAAACCATTCAAGAGTTTGAAGATTATGTTCAATCTGTGGACATTGCTGCATTCA
ACAAAATTTAA

elongation factor 1-beta（EF-1β）(Sequencing results)

AACTACTTGAGCCAGAAAAGTTACATTGTTGGATATGAACCATCCAAAGCCGACGTTG
ATACTTTTGCTGCGATTCAAGCACCCCAGGCCAAGACTCCTAATGTGCTCAGGTGGTAC
AACCACATCAAGTCGTTCACTGAGAAAGAGCGCACCCAGTTCCCAAACAAAAAAGCT
GAGTTTTCAGTGTCTTCGGCTAATGCTGCAAAACCGGCAGATGACGATGATGACGATGT
AGATTTGTTCGGTTCTGATGATGAAGATGATGAAGAAGCAGAACGTATCAAACAAGAA
AGACTAAAGGCTTATGCTGAAAAGAAAGCCACCAAAAAAGTAATTATCGCCAAGAGTA
GCATTGTACTTGATGTTAAACCATGGGATGATGAAACCGATATGAAACAATTAGAAACA
CAAGTCCGATCCATTAACATGGATGGTTTAGTGTGGGGAGCTTCAAAATTAGTCGAGAT
TGCTTTTGGTATCAAGAAGCTTCAAATTATGTGTATTGTAGAAGATGATAAAGTATCTGT
CGATGCTCTTACAGAAACCATTCAAGAGTTTGAAGATTATGTTCAATCTGTGGACATC

Sequence comparison results

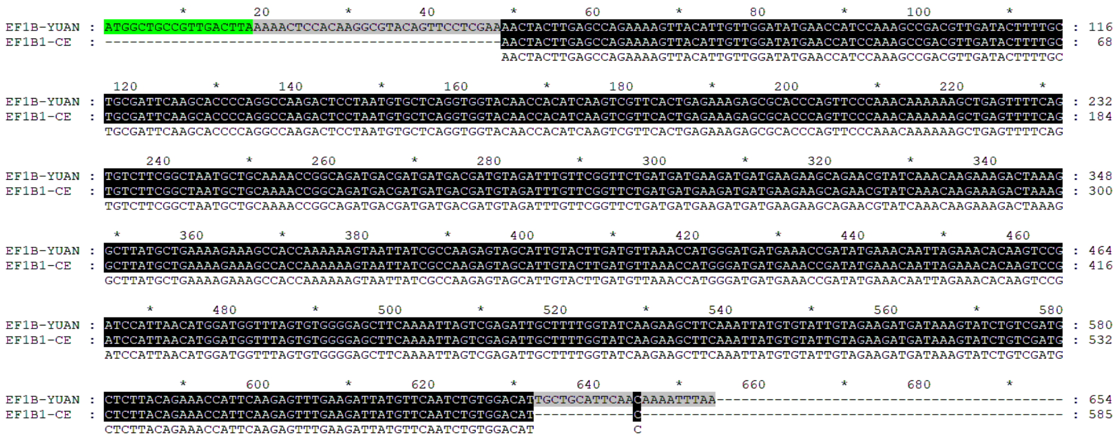

5. Glyceraldehyde-3-phosphate (GAPDH) (original sequence)

>XM_025343821

ATGTCGAACATTGGTATCAACGGATTTGGCCGTATTGGTCGATTGGTTTTAAGAGCTTCA
TTGGAAAAGGGCGCCAAAGTTGTAGCCATCAATGACCCATTCATTGGTATTGAATATAT
GGTATACTTGTTCAAGTATGATTCTACTCATGGACGTTTCAAGGGCGAAGTTTCTGTTGA
TGGAGATTTTCTTATCGTCAATGGAAACAAAATCAAGGTGTTCTCTGAACGCGACCCTA
AAGCCATCCAATGGGGATCTGCTGGTGCCGATTACGTTGTTGAATCCACCGGTGTATTT
ACCACCATTGAAAAAGCCTCTGCTCATTTGGAAGGTGGAGCCAAGAAAGTTATCATCT
CTGCACCAAGTGCTGATGCACCAATGTTTGTTGTTGGTGTTAATTTGGATGCTTACAAT
CCATCATTCAAAGTTGTATCTAATGCTTCTTGCACAACTAACTGCTTGGCTCCATTAGCC
AAGGTTATTCATGACAACTTTGGAATCATTGAAGGTCTTATGACTACTGTTCATGCTACT
ACTGCCACTCAAAAAACTGTTGATGGGCCATCTGGAAAATTGTGGAGAGATGGTAGAG
GTGCTGCCCAAAACATCATCCCAGCATCTACTGGAGCAGCTAAGGCTGTTAGTAAAGT
CATCCCAGAACTTAATGGAAAATTAACTGGAATGGCTTTTAGAGTACCAGTTGCTAATG
TTTCTGTTGTTGACTTGACTGTAAGACTTGCAAAACCAGCATCCTACCAAGATATCAAA
GATAAGGTCAAGGAAGCAGCAGCAGGACCATTGAAAGGAATTTTGGGTTATACTGAAG
ATGAAGTTGTGTCTTCTGACTTTATTGGTGATACCCATTCATCGATCTTTGACGCTAAGG
CGGGAATTTCATTGAACGATCAATTTGTCAAGCTTATCTCATGGTACGACAATGAATATG
GTTATTCCAATCGTGTTGTGGACTTAATCAAGTA
CATGCAATCTAAGGATTAA

Glyceraldehyde-3-phosphate (GAPDH) (Sequencing results)

TCTTGTTCAGTACGATTCTACTCATGGACGTTTCAAGGGCGAAGTTTCTGTTGATGGAG
ATTTTCTTATCGTCAATGGAAACAAAATCAAGGTGTTCTCTGAACGCGACCCTAAAGCC
ATCCAATGGGGATCTGCTGGTGCCGATTACGTTGTTGAATCCACCGGTGTATTTACCAC
CATTGAAAAAGCCTCTGCTCATTTGGAAGGTGGAGCCAAGAAAGTTATCATCTCTGCA
CCAAGTGCTGATGCACCAATGTTTGTTGTTGGTGTTAATTTGGATGCTTACAATCCATCA
TTCAAAGTTGTATCTAATGCTTCTTGCACAACTAACTGCTTGGCTCCATTAGCCAAGGTT
ATTCATGACAACTTTGGAATCATTGAAGGTCTTATGACTACTGTTCATGCTACTACTGCC
ACTCAAAAAACTGTTGATGGGCCATCTGGAAAATTGTGGAGAGATGGTAGAGGTTGTT
GCCAAATA

Sequence comparison results

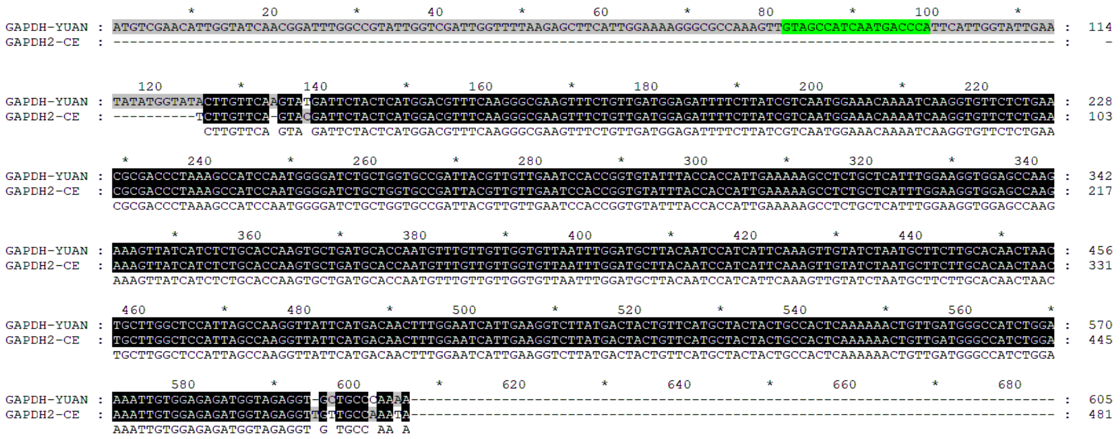

6. Heat Shork Protein 70　(HSP70) (original sequence)

>XM_025350978.1（HSP70)

ATGGTCGGAAAGACTGCTATCGGTATCGACCTGGGCACCACCTATTCTTGTGTGGGCGT
CTGGCAACACGGAAAAGTGGAGGTAATAGCCAACGATCAAGGAAACAGGACCACGCC
AAGTTATGTGGCGTTCACGGACACGGAACGGTTGATCGGGGACGGAGCCAAGAACCA
GGTGGCGATGAACCCGGTGAACACCGTGTTCGACGCTAAACGTTTGATCGGACGTCGG
TTTGACGACGAGAAGACACAAGCGGACATGAAACAATGGCCGTTCAAAGTGGTGAAC
GACTGCGGAAAGCCCAAAATCCAGGTTGAATTCAAAGGCGAGCGGAAAGTGTTCGCG
CCGGAAGAAATTAGTTCGATGGTGCTGACGAAAATGAAGGAGACCGCGGAAGCGTAC
CTGGGCCGTAGCGTGACGGACGCGGTGATCACGGTGCCGGCGTACTTCAACGATTCGC
AGAGACAGGCGACGAAGGACGCGGGCGCGATAGCCGGGCTGAACGTGATGCGGATAA
TCAACGAACCGACAGCCGCGGCCCTGGCGTACGGCCTAGACAAGAACCTGAAAGGCG
AGAGGAACGTATTGATATTCGATCTGGGCGGCGGTACGTTCGACGTGTCGGTTCTGCAG
ATCGACGAGGGTTCGATATTCGAAGTGAAATCGACGGCTGGCGACACGCACTTGGGCG
GCGAAGACTTCGACAACCGGCTGGTGAGTCATCTGGCCGACGAGTTCAAGAGGAAGT
TCAAAAAAGACGTGCGTAGCAATCCGAGGGCGCTGAGACGGCTACGGACGGCCGCCG
AGCGGGCCAAGAGAACGTTGTCGTCCAGCTCGGAGGCGACCATTGAGATCGACGCCC
TGATGGAAGGCGTCGATTTCTACACGCGAGTCTCCCGGGCCCGTTTCGAGGAGTTATG
CGCGGACCTGTTCAGATCGACCCTGCAACCGGTTGAAAAGGCTTTGGCGGACGCCAA
GTTGGACAAGGGAGACATCCACGACATTGTGCTGGTGGGCGGTTCGACAAGGATCCC
GAAGATCCAAAGTCTGCTGCAAAACTTTTTCTGCGGCAAGCCTCTCAACCTGTCCATC
AACCCGGACGAAGCCGTGGCTTACGGCGCCGCGGTGCAGGCGGCCATCCTGAGCGGT
GACACGAGTTCCGCGATCCAAGACGTGCTGCTCGTGGACGTTACGCCCCTGTCGTTGG
GCATCGAGACCGCGGGCGGAGTGATGACGAAAATCGTCGAGCGCAATTCCACCATCCC
GTGCAAACAAACGCAAACGTTCACGACGTACGCGGACAACCAACCGGCCGTCACCAT
TCAGGTGTTCGAAGGAGAAAGAGCAATGACCAAGGACAACAATCTGTTGGGAATATTC
GACCTCACCGGCATACCTCCGGCGCCCAGGGGTGTGCCCAAGATCGAGGTGACTTTCG
ACATGGACGCCAACGGAATTTTGAACGTGTCGGCCAAAGACAACAGTTCCGGCCGCT
CCAAGAACATCGTCATCAAGAACGACAAGGGTCGACTGTCTCAAGCCGAAATCGATC
GTATGCTCAGCGAGGCCGAACGGTACAAAGAAGAGGACGAACGGCAAAAGGCCAAG
ATCGCGGCCAAGAACCAGTTGGAGAGCTACGTGTTCGGCGTGAAACAAGCGTTGGAC
GAAGCCGGTGACAAGTTGACCGAATCTGAGAAGAACACTGGCAAACAAGAATGCGAC

GCAGTCGTCCAGTGGTTAGACAACAATCAATTGGCAGACAAAGAAGAGTACGAGTATA
AACTGAAGGAAATACAAAAGAGTTGCTCTGCTCTCATGATGAAGATACACGGCGGTGG
ACAACCCGGCGGTGCGCCTCCCGGTGCCCAAGGTTTCCCGGGTTCTAGAGGAGGACCT
ACCGTCGAAGAAGTCGATTAA

Heat Shork Protein 70 (HSP70) (Sequencing results)

CTGGCACTATTCTTGTGTGGGCGTCTGGCACACGGAAAAGTGGAGGTAATCGCCAACG
ATCAAGGAAACAGGACCACGCCGAGTTATGTGGCTTTCACGGACACGGAACGGTTGAT
CGGGGACGGAGCCAAGAACCAGGTGGCGATGAACCCGGTGAACACCGTGTTCGACGC
CAAACGTTTGATCGGACGTCGGTTTGACGACGAGAAGACACAAGCGGACATGAAACA
ATGGCCGTTCAAAGTGGTGAACGACTGCGGAAAGCCCAAAATCCAGGTTGAATTCAA
AGGCGAGCGGAAAGTGTTCGCGCCGGAAGAAATTAGTTCGATGGTGCTGACGAAAAT
GAAGGAGACCGCGGAAGCGTACCTGGGCCGTAGCGTGACGGACGCGGTGATCACGGT
GCCGGCGTACTTCAACGATTCGCAGAGACAGGCGACGAAGGACGCGGGCGCGATAGC
CGGGCTGAACGTGATGCGGATAATCAACGAACCGACAGCCGCGGCCCTGGCGTACGG
CCTGGACAAGAACCTGAAAGGCGAGAGGAACGTGTTGATATTCGATCTGGGCGGCGG
TACGTTCGACGTGTCGGTTCTGCAGATCGACGAGGGTTCGATATTCGAAGTGAAATCG
ACGGCTGGCGACACGCACTTGGGCGGCGAAGACTTCGACAACCGGCTGGTGAGTCAT
CTGGCCGACGAGTTCAAGAGGAAGTTCAAAAAAGACGTGCGTAGCAATCCGAGGGCG
CTGAGACGGCTGCGGACGGCCGCCGAGCGGGCCAAGAGAACGTTGTCGTCCAGCTCG
GAGGCGACCATTGAGATCGACGCCCTGATGGAAGGCGTCGATTTCTACACGCGTGTCT
CCCGGGCCCGTTTCGAGGAGTTGTGCGCGGACCTGTTCAGATCGACCCTGCAACCCGG
TGGAAAAGGCTTTGGCCGGACGCCAAGTTTGGACAAGGAGTCTCCCCCCCGCCCTCTT
GTTTG

Sequence comparison results

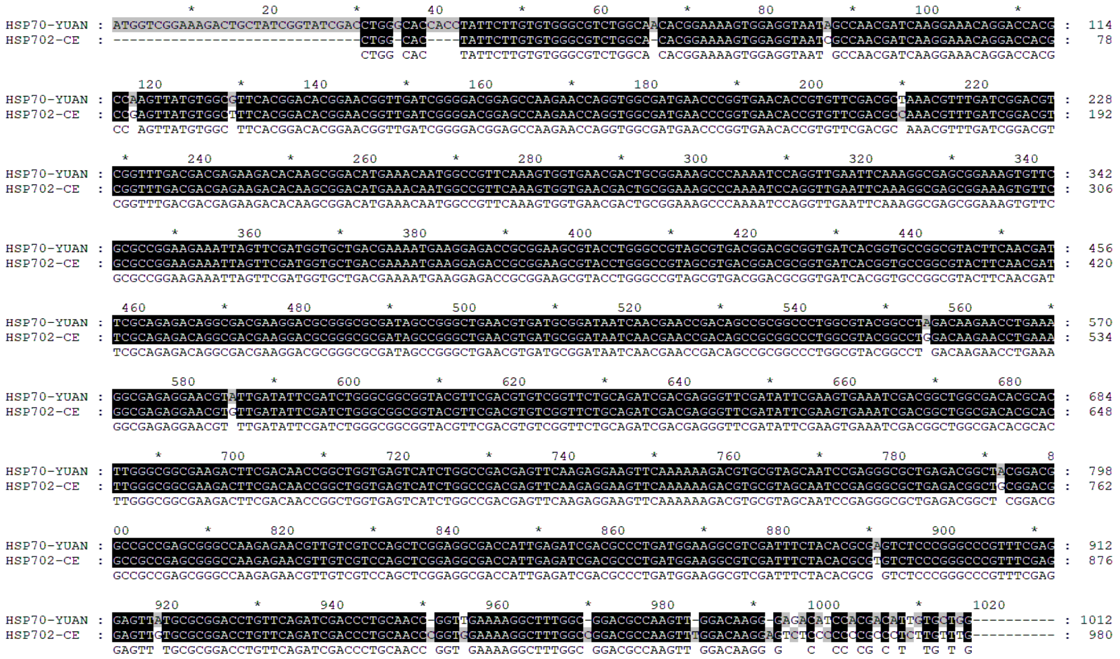

7. Ribosomal protein L 7 （RPL7) >XM_025345708.1 (RPL7) (original sequence)

ATGGTTGATGACAATGCCCCCAAAAGGCTTCCGGCGGTGCCGGAGTCTGTACTCAAAA
GACGTAAGGCTCGTACAGCATTCAAACTTAAATCATTGAAGAAAGCTATTGAGGAACG

TAAAGAACGCGTGAAAAAAACTAAGAAGTATTTTAAACGCGCTGAAGCTTATGTTAAG
GAATTTAGAATGAAGGAAAGAGATGAAATCCGTTTAGCAAGGAATGCTAAAAAAGCTG
GTGATTTTTATATTCCTCCTGAACCAAAATTAGCATTCATCATCCGTATTCGTGGTGTGAA
TCAAGTGGCTCCTAAAGTGAAAAAAGTATTGCAACTGTTCAGATTACGTCAGATCAAC
AATGGAATATTTATCAAATTAAACAAAGCAACATTAAATATGTTGAGGATTTGTGAACCA
TATGTAACTTGGGGATATCCTAACTTGAAGAGTGTAAGGGAATTGGTCTACAAAAGAG
GATTTGCCAAAATCAAGGGTCAACGCATTCCGATCACTAACAATGAAATGATTGAAAA
GAAATTGGGAAAATATGGTATCATATGCACGGAAGATTTAGTTCATTGTATATACAGAGC
TGATCGTCGATTCAAGTATGCCATGAACTTCCTTTGGCCTTTCAAATTAAACACCCCAA
CTGGAGGCTGGCGTAAGAAGACCAATCACTATGTTGAAGGTGGAGATTTTGGAAACCG
TGAAGATACCATTAACAGATTATTGAGAAAAATGGTTTAA

Ribosomal protein L 7 (RPL7) (Sequencing results)

GAGAGCTATTGAGGACGTAAAGAACGCGTGAAAAAAACTAAGAAGTATTTTAAACGC
GCTGAAGCTTATGTTAAGGAATTTAGAATGAAGGAAAGAGATGAAATCCGTTTAGCAA
GGAATGCTAAAAAAGCTGGTGATTTTTATATTCCTCCTGAACCAAAATTAGCATTCATCA
TCCGTATTCGTGGTGTGAATCAAGTGGCTCCTAAAGTGAAGAAAGTATTGCAACTGTTC
AGATTACGTCAGATCAACAATGGAATATTTATCAAATTAAACAAAGCAACATTAAATATG
TTGAGGATTTGTGAACCATATGTAACTTGGGGATATCCTAACTTGAAGAGTGTAAGGGA
ATTGGTCTACAAAAGAGGATTTGCCAAAATCAAGGGTCAACGCATTCCGATCACTAAC
AATGAAATGATTGAAAAGAAATTGGGAAAATATGGTATCATATGCACGGAAGATTTAGT
TCATTGTATATACAGAGCTGATCGTCGATTCAAGTATGCCATGAACTTCCTTGGGCCTTT
TAAA

Sequence comparison results

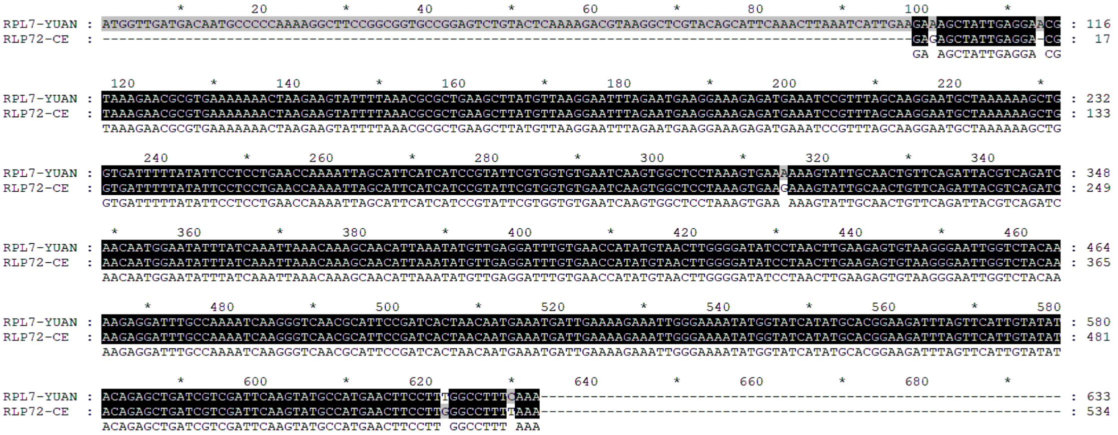

8. TATA box binding protein (TATA) (original sequence)

>XM_025347315.1 (TATA)

ATGGATCAAATGTTACCGAGTCCTGGGTTTAGTATTCCTAGTATTGGAACCCCAGTACAT
CAACCAGAAGAAGATCAACAGATTATGCAAATGCCACCGCTACAGCCACAAGGTCTAG
CTATGAGTACTAGTAGTCTACCAACTCAACAGTCGATGAATAAAATATATCAGACAAAT
AATATGGGCTACGCTACGCCACACAGCATGATGCATTCACAAACACCTGTGCATCAAAG
TATGCCATCTTTGACATCCATCAGTTCATCATCATCGACTATACCAGCGCTACAAACTATT
CAAAACCCAGCTACGCCTGCCCCGATGACACCTATGACACCAGCTTCTGCTGATCCTG
GGATTGTACCTCAACTCCAGAATATAGTGTCTACAGTAAATCTTGGATGTCGGCTTGATC
TTAAAACTATTGCTTTACATGCACGAAATGCTGAATTTAATCCAAAACGATTTGCTGCTG

TTATAATGAGGATACGTGAACCACGTACTACAGCTTTAATCTTTAGTTCTGGTAAAATGG
TATGCACTGGCGCAAAAAGTGAAGAAGATTCTAGACTGGCAGCAAGAAAATATGCTAG
GATCATTCAAAAGCTTAGTTTTCCAGCAAAGTTCTTAGACTTTAAAATTCAAAACATGG
TTGGCAGTTGTGATGTAAAGTTTCCTATACGGCTTGAGGGATTGGTACTCACTCATGGT
CAATTTAGCAGTTATGAACCAGAATTGTTTCCTGGACTTATTTACAGAATGGTTAAACC
ACGTATTGTATTACTCATTTTTGTATCTGGTAAAGTTGTACTGACAGGTGCCAAAGTACG
TCAAGAAATTTATGATGCTTATGATAATATTTATCCAATATTGAAAAGCTTTAAAAAAAA
CTAA

TATA box binding protein (TATA) (Sequencing results)

ACTGTTTGGACCCCAGTACATCAACCAGAAGAAGATCAACAGATTATGCAAATGCCAC
CGCTACAGCCACAAGGTCTAGCTATGAGTACTAGTAGTCTACCAACTCAACAGTCGATG
AATAAAATATATCAGACAAATAATATGGGCTACGCTACGCCACACAGCATGATGCATTCA
CAAACACCTGTGCATCAAAGTATGCCATCTTTGACATCCATCAGTTCATCATCATCGACT
ATACCAGCGCTACAAACTATTCAAAACCCAGCTACGCCTGCCCCGATGACACCTATGAC
ACCAGCTTCTGCTGATCCCGGGATTGTACCTCAACTCCAGAATATAGTGTCTACAGTAA
ATCTTGGATGTCGGCTTGATCTTAAAACTATTGCTTTACATGCACGAAATGCTGAATTTA
ATCCAAAACGATTTGCTGCTGTTATAATGAGGATACGTGAACCACGTACTACAGCTTTA
ATCTTTAGTTCTGGTAAAATGGTATGCACTGGCGCAAAAAGTGAAGAAGATTCTAGACT
GGCAGCAAGAAAATATGCTAGGATCATTCAAAAGCTTAGTTTTCCAGCAAAGTTCTTAG
ACTTTAAAATTCAAAACATGGTTGGCAGTTGTGATGTAAAGTTTCCTATACGGCTTGAG
GGATTGGTACTCACTCATGGTCAATTTAGCAGTTATGAACCAGAATTGTTTCCTGGACTT
ATTTACAGAATGGTTAAACCACGTATTGTATTACTCATTTTTGTATCTGGTAAAGTTGTAC
TGACAGGGCCC

Sequence comparison results

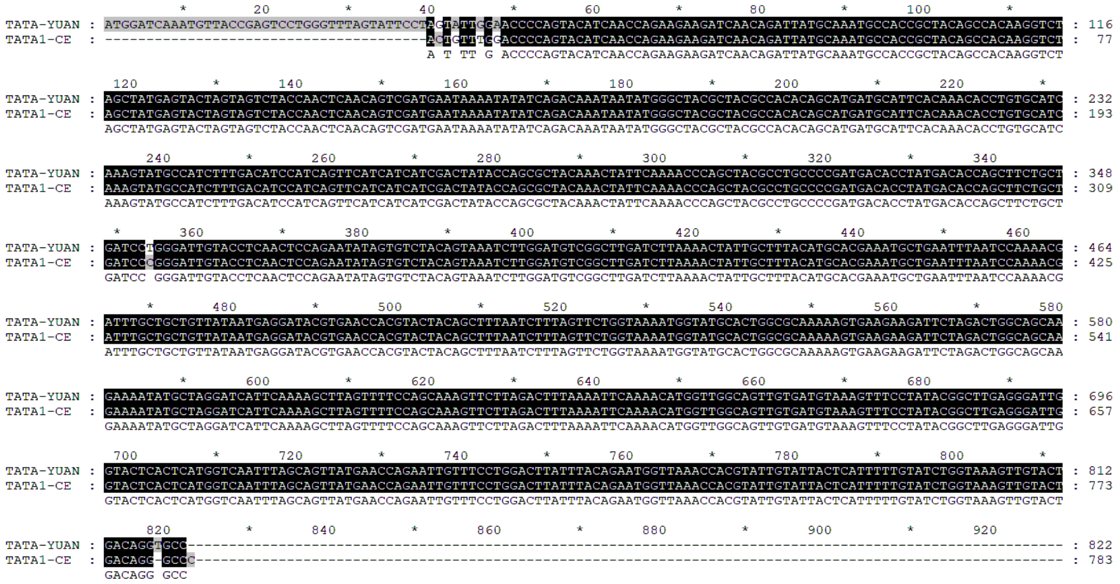

9. 28S ribosomal (28S) (original sequence)

>XM_025343486.1

ATGGGAAGAAAAGCGAAATTCGGCGATGAAGGAGCAGCTAAACAAATAAAAGGTCCT
GGACGAAAAGCAAAAAAACAAAAGCCACCAAAAATGCTACCAGGACTTCCAAAACT
CGAAGGAGATAATGAGCCAAAAAAATTAAGTCATCGACAAAAACAGCGTGCTTCTAGA

AGAATGAAAAAAAAAGAAGAAAGAAAGTTAATAAAAAAAGAAAAAAAGAAAGGTGT
TAAAAACGTTGATGATGTCGAAAATAAAAAGGATGAATCTGTTACTACTAAGAACATAA
AAAATGAAATTGTTAAACAATTTTCTGATAGTAATAGTAGTTGGTTGAAATCAAAAAAC
TCAAAATCAATTATTCCTGAAAAAGATGATGATATAGAAAGTTATGACAGTAATTCAGAT
ATAGATGATGAAATAACAGAGGAAAATGGTTTAGAAAGTTTTGATGAAGATGGCCTATC
AGATGATGATGACTATTTGGCGGGTACATTGGATGATGTTGATAGTTCAAACGAATCTG
GAATTGAAGATACTACTATTAAAAAGAAAAAAACTTCTACAAAGAAAAGAAAAGCAG
AAGAAAGTGATGACGAATTATTACCTATTGAGAAAGCAGCTAAAAAATTAAAAGCTGC
AAAGAAAAAAGAAGACGCTGAAGCCAATGAGGAAATGAAAATGCAAATTAACGTTGC
AAGCCAAGATGTTTTTGTTTTTCCTGCCAGTGATAAAATTGATGAACATATACCTATACC
TGAAGTAGAACAAAGAATTAAAGATATATTATTAGTATTATCTAATTTCAATAAATTTCGG
GAGGAAAATCGAAGCCGACAAGAGTACACTGATTTATTACTCAAGGATTTATGTACCTA
TTTTAGTTATAACACATTTTTAATGGAAAAAATGATGCATTTGTTTCCCCTAGAAGATTT
AATGTCTTTTTTAGAAGCCAGTGAAACGCCTAGACCAGTAACTATTCGAACAAATAGTT
TGAAAACTAGGAGACGTGACCTTGCTCAAGCATTGATTAACCGGGGAGTAAATTTGGA
TCCTATTGGTAATTGGTCTAAGGTAGGGCTTGTTGTATACAATTCTACAGTACCAATAGG
AGCAACTCCTGAATATCTGGCTGGACATTATATGCTTCAAGCAGCTTCCAGTATGTTACC
TGTTATGGCGTTAGCTCCTCAAGAAAATGAATTCATTTTGGATATGTGTTCTGCACCTGG
TGGTAAAGCTTCACATATTGCTGCTATAATGAAAAATACAGGAGTTTTAATAGCTAATGA
TGTAAATAAAAACCGTGCAAAGGCAGTAATTGGTAATTTTCATAGAATGGGAATAGCCA
ATTCTGTTATTTCAACATATGATGGTAGACATATACCTCAATTATTCAAAAACTTTGATAG
AGTATTACTAGATGCTCCTTGTACTGGAACTGGAGTTATAAGTAAAGATTCTGGAGTTA
AAATGAGTAAAGATGAAACTGATTTGCAAAGATGTTTTACATTACAGCGAGAATTATTG
CTTGCTGCTATAGATTCGTTGAATTTTAAGAGTTCAACAGGCGGTTATTTAGTTTATTCG
ACTTGTTCAGTACTTGTAGAAGAAAATGAATGTGTGATAGATTATGCTTTAAAAAAGAG
GGATGTAAAACTAGTTGATACAGGATTAAGTTTTGGTACTGAAGGTTTTACAAATCTTC
GTCAACACAGGTTTCACCCTACAATGAAACTTACAAGGCGTTTTTACCCGCATACTCAT
AATATGGATGGATTTTTTGTTGCAAAACTAAAAAAATTTTCAAATATTATTCCCAAAAAT
AATGAATCTGAAGATAATGAAGATGAAAATGATTTAAATAATGTTGATGCAGAAAATGC
AGAGGTTCAAGAAACTATTGAAAAACCAGTAGCAAATATAAAAAAACCAAAACATGG
GCGTGGCAAAAACAGAGTTGAAAAAAAATAA
28S ribosomal（28S）(Sequencing results)
TGCTAAATGGGTCTCGCTGGGGCGAAAGCAAAAAGCAAAGCACAAAAATGCTACAGA
CTGCAAAGCTCGAAGAGATATGAGCAAAAAAACTGAAGTCATCGACAAAAGCAGCGG
TGCTCTAGAGAATGAAAAAAAAAGAGAAAGAAAGTATAAAAAAAGAAAAAAAGAAAG
GTGTAAAAACGTTGATGATGTCGAAAATAAAAAGGATGAATCTGTTACTACTAAGAACA
TAAAAAATGAAATTGGTAAACAATTTTCTGATAGTAATAGTAGTTGGTTGAAATCAAAA
AACTCAAAATCAATTATTCCTGAAAAAGATGATGATATAGAAAGTTATGACAGTAATTC
AGATATAGATGATGAAATAACAGAGGAAAATGGTTTAGAAAGTTTTGATGAAGATGGC
CTATCAGATGATGATGACTATTTGGCGGGTACATTGGATGATGTTGATAGTTCAAACGAA
TCTGGAATTGAAGATACTACTATTAAAAAGAAAAAAACTTCTACAAAGAAAAGAAAAG
CAGAAGAAAGTGATGACGAATTATTACCTATTGAGAAAGCAGCTAAAAAATTAAAAGC
TGCAAAGAAAAAAGAAGACGCTGAAGCCAATGAGGAAATGAAAATGCAAATTAACGT
TGCAAGCCAAGATGTTTTTGTTTTTCCTGCCAGTGATAAAATTGATGAACATATACCTAT

ACCTGAAGTAGAACAAAGAATTAAAGATATATTATTAGTATTATCTAATTTCAATAAATTT
CGGGAGGAAAATCGAAGCCGACAAGAGTACACTGATTTATTACTCAAGGATTTATGTAC
CTATTTTAGTTATAACACATTTTTAATGGAAAAAATGATGCATTTGTTTCCCCTAGAAGA
TTTAATGTCTTTTTTAGAAGCCAGTGAAACGCCTAGACCAGTAACTATTCGAACAAATA
GTTTGAAAACTAGGAGACGTGACCTTGCTCAAGCATTGATTAACCGGGGAGTAAATTT
GGATCCTATTGGTAATTGGTCTAAGGTAGGGCTTGTTGTATACAATTCTACAGTACCAAT
AGGAGCAACTCCTGAATATCTGGCTGGACATAAGCAGCCCGGATA

Sequence comparison results

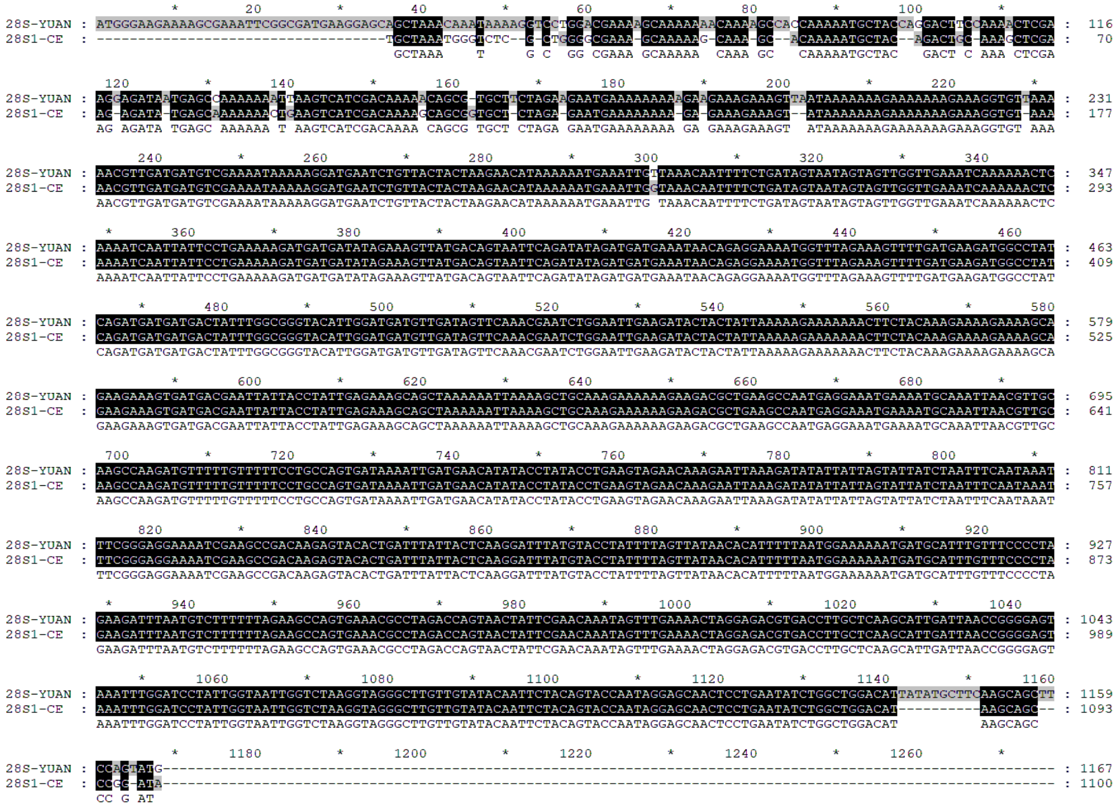

Supplement: Supplementary file 1 [file insects-15-00522-s001.zip › insects-3070081-supplementary.pdf]
